# Attitudes to Spiritual Care Among Nursing Staff in an Oncology Clinic in 2003 and 2023: A Longitudinal Quantitative Case Study

**DOI:** 10.1155/jonm/5613893

**Published:** 2026-09-29

**Authors:** Mikael Lundmark, Sara Sjöstedt de Luna

**Affiliations:** ^1^ Department of Historical, Philosophical and Religious Studies, Umeå University, Umeå, Sweden, umu.se; ^2^ Department of Mathematics and Mathematical Statistics, Umeå University, Umeå, Sweden, umu.se

## Abstract

**Aim(s):**

To explore the attitudes to spiritual care among nursing staff at one Swedish oncology clinic, with special focus on determinants of such attitudes and their stability over time.

**Design:**

The design of the study is a quantitative, longitudinal and comparative design.

**Methods:**

A questionnaire‐based replication study conducted in 2003 and 2023, using statistical methods. Suggested influencing factors to spiritual care and indicators for attitudes to spiritual care were identified in studies prior to the 2003 study.

**Results:**

The staff expresses positive attitudes to spiritual care in both studies. More nursing staff considered patients’ spiritual needs a concern in 2023. There were no big differences in self‐estimated frequencies of how much the colleagues provide spiritual care, the self‐estimated ability to provide spiritual care, or the self‐estimated frequency to provide spiritual care. Education in spiritual care was considered important in both studies, but the received education in spiritual care was perceived as decreased in 2023. Several of the influencing factors of 2003 have lost their relevance in 2023 but ‘nonorganized religiousness’ and ‘belief in God’ are remaining stable factors over time. The factor ‘organized religiousness’ significantly correlates with the indicator ‘self‐estimated ability to provide spiritual care’ in 2003, whereas ‘nonorganized religiousness’ significantly correlates with the same indicator in 2023.

**Conclusions:**

The results indicates that what might influence attitudes to spiritual care changes over time and can depend on societal changes.

**Summary**
• Implications for the profession and/or patient care.
◦ When studying determinants of attitudes to spiritual care, the determinants’ stability over time should be accounted for.
• Impact.
◦ The attitudes to spiritual care in one Swedish oncology clinic and whether determinants to such attitudes change over time.
◦ Overall positive attitudes to spiritual care, small changes have occurred, and some determinants are not stable over time.
• Reporting Method:
◦ STROBE.
• Patient or Public Contribution:
◦ None.

## 1. Introduction

This longitudinal study investigates attitudes to spiritual care at an oncology clinic in 2003 and 2023 to provide insight into whether such attitudes are sensitive to, for example, societal changes related to spirituality. As such, the study will benefit nursing management by increasing the understanding of the relationship between clinical practice and the broader societal context in which it takes its place. This investigation also provides valuable insights to take into account when implementing or evaluating studies on spiritual care in other healthcare contexts and other parts of the world.

The importance of spirituality for health, at least when related to the practice of a religion (what Schnell [1] calls spirituality with religion, in contrast to spirituality without religion), has been established in numerous studies [2]. The implications for this in nursing contexts have been the subject of increased research interest over the last forty years [3], especially so in the domain of palliative care research, where spirituality, according to Lundberg et al., ‘*is often seen as something “enigmatic,” yet inherently human and natural’* (p. 7) [4]. Furthermore, it is assumed that every person has a spiritual dimension and that healthcare professionals are expected to be able to provide spiritual care [4]. Similar views are expressed in studies addressing other areas of nursing, such as critical care [5], mental health nursing [6], general practice nursing [7] and nursing home care [8]. Recurring themes in research on spirituality and healthcare include how the concepts of ‘spirituality’ [9] and ‘spiritual care’ [10] are understood, as well as what is suitable competences for spiritual care [11] and what determines ‘spiritual care practices’ [12].

Several conceptual and methodological problems associated with research on spirituality and health have been noted [13]. Regarding research on determinants of spiritual care practices, concerns have been raised with respect to the impact of cultural differences on spiritual care practices [12]. Notwithstanding this body of previous research, one question that has not been addressed is whether such determinants remain stable over time or whether they are sensitive to, for example, societal changes.

To investigate this question, a study was conducted in 2023 that replicated a study made 20 years earlier (i.e., in 2003) that investigated the understanding of, attitudes to and perceived difficulties associated with spiritual care among nursing staff in a holistic nursing context at a Swedish oncology clinic [14–16].

At the turn of the millennium, a strong connection existed between spiritual care and holistic health philosophy [15, 16], that is, ‘*a philosophy of healthcare practice that accounts for the human being as a whole and in interaction with the environment, considering not only the physical needs but also psychological, emotional, social, economic and spiritual needs of the patient*’ ([17], p. 55). As such, a holistic health philosophy serves as a theoretical framework through which spiritual care becomes an integrated part of healthcare [17]. The decision to do the study on an oncological clinic was informed by a basic methodological assumption borrowed from Wiliam James’ research in the psychology of religion called ‘the method of the extreme cases,’ according to which a few extreme cases can provide good general knowledge about most nonextreme cases [18]. It was assumed that an oncology clinic can be regarded as ‘extreme’ in this respect because of the quite attendant presence of palliative elements and the fact that the patients’ average stay at the clinic was quite long. These circumstances would allow the patients and the clinic’s staff to get to know each other well and building up personal confidence between staff and the patients. Furthermore, it was argued that the general uncertainty about their future or certainty about their impending death that many of the clinic’s patients experienced would create conditions conducive to spiritual and existential reflections. Such reflections could also be expected to be expressed in conversations between the nursing staff and their patients [16, 19, 20].

The tentative definition of the concept of ‘spiritual care’ used in the 2003 study was informed by the results of Strang et al.’s [21] study of how Swedish nursing staff at seven different healthcare units characterize their patients’ spiritual needs and verbatim reused in the 2023 study. It runs as follows:

“making possible/facilitating for the patient, with the help of suitable nursing interventions, to express and discuss existential questions and to practise his/her spirituality (which may be done through the practising of a specific religion but also through activities which do not need to be of religious nature).” ([16], p. 868).

The results are discussed with particular reference to examples of societal changes related to the Swedish spiritual climate and some identified educational changes regarding spirituality and health in the academic education of nurses since 2003.

### 1.1. Societal Changes Related to Spirituality and Educational Changes Regarding Spirituality and Health in the Academic Education of Nurses Since 2003

During the 20 years that have passed since Lundmark conducted his study in 2003, Swedish society has undergone many changes [22], including changes in the country’s spiritual climate. When comparing Wave 4 (which covers the years 1999–2003) with Wave 7 (which covers the years 2017–2022) in the World Values Survey (WVS), it is noted that in both surveys, Sweden achieved the highest factor scores in the domain of ‘self‐expression values’ [23, 24], with an increase between the two waves from 2.8080 to 3.1133. ‘Self‐expression values’ reflect a humanistic and emancipative ethos where human autonomy and individual choice are emphasized [25]. However, as Thurfjell [26] has argued, ‘self‐expression values’ are relevant to understanding aspects of Swedish spirituality. For example, the trend in Swedish society toward an ever‐increasing sense of individualization and an increased privatization of spirituality is parallelled by an increasing diversity in spiritual expression [26]. Sweden also scored high in terms of ‘secular values.’ In the period between Wave 4 and Wave 7 in the WVS, the Swedish population’s reported that *belief in God* decreased from 46.6% to 34.4%. The proportion of the population who reported they *do not believe in God* increased from 40.6% to 60.8%. A similar trend can be observed concerning *belief in life after death* and *participation in religious meetings* [23, 24].

In a review of how spiritual care is addressed in various written sources in Sweden, Lundmark [27] shows a mixed picture of how spiritual care has been addressed during the time period in question. The sources of Lundmark [27] are related to healthcare in general and palliative care in particular (“competence descriptions” issued by the National Board of Health and Welfare (Swedish: Socialstyrelsen), and the Swedish Nurses’ Association (Swedish: Svensk sjuksköterskeförening); different editions of the Swedish Handbook for Health Care (Swedish: Vårdhandboken, an online resource available for all healthcare staff) as well as Swedish textbooks are used in academic nursing programs.

In the Swedish healthcare system, there is basically two different types of nurses: nursing auxiliaries and registered nurses. A nursing auxiliary 2023 usually had the title nursing assistant (Swedish: undersköterska) with a secondary education (Swedish: gymnasieutbildning) in nursing. In 2003, a nursing auxiliary could also have had a shorter education in nursing and was then titled healthcare assistant (Swedish: sjukvårdsbiträde). Registered nurses (Swedish: Sjuksköterska) has at least 2‐3 years’ university education in nursing (depending on which time period the education was conducted). They could also be further trained to a specialist nurse. The competence descriptions on registered nurses are quite similar during the time period in the sense that they do address patients’ spiritual needs as a part of what registered nurses competencies should include. Similar competence descriptions for nursing assistants were first issued as late as 2021 in which spirituality is not mentioned [27].

Spiritual care is given relatively much attention in Swedish textbooks on palliative nursing printed in the beginning of this millennium, whereas they are given very little or no attention at all, in similar textbooks printed recently. One exception here is the introductory textbook commonly used by Swedish education programs for registered nurses, which is not a specialised textbook on palliative care, addressing spiritual care at a chapter’s length. In more recent textbooks, the concept of ‘spirituality’ seems to be consequently replaced with the concept ‘existential.’ In the Swedish handbook for healthcare, the ‘spiritual/existential’ dimension is mentioned as a part of what palliative care should address in the 2016 online edition whereas in the 2024 online edition, ‘spiritual’ has disappeared and only ‘existential’ remains [27].

## 2. The Study

### 2.1. Aims and Research Questions

This article is based on some of the results of the replication study conducted in 2023 on Lundmark’s 2003 study. The following aims and research questions (RQs) are addressed:

 Aim 1: To replicate the 2003 study by responding to the following RQs (using similar RQs as in 2003 and with an almost identical questionnaire):
   (RQ1) What are the attitudes to spiritual care in a Swedish holistic nursing context?
   (RQ2) What are the attitudes to the role held by oneself and the other members of the nursing staff with regard to spiritual care?
   (RQ3) To what extent do nursing staff members believe that they have received education in spiritual care?
   (RQ4) What is the relation between the nursing staff’s attitudes to spiritual care and the following factors:
    a. Education in spiritual care
    b. Self‐reported spirituality
    c. Professional position (registered nurse or nursing auxiliary)
    d. Number of years working in healthcare
    e. Sentiments of being at ease/not at ease while providing spiritual care
 Aim 2: To perform a comparative analysis of the results of the 2003 study with the results of the 2023 study, to identify similarities and differences. This aim is informed by the RQ:
   (RQ5) Have attitudes to spiritual care among nursing staff in a Swedish holistic nursing context, represented by an oncological clinic, changed between 2003 and 2023? If so, how have attitudes changed?

### 2.2. Operationalization of Concepts

In the 2003 study, Lundmark identified, through a literature review, several determinants or, as Lundmark chose to call it: *factors* that have been suggested to influence attitudes to spiritual care. These factors include (i) professional position (registered nurse or nursing auxiliary); (ii) perceived educational experience in spiritual care; (iii) self‐reported spirituality (belief in God, belief in life after death, and organized and nonorganized religiousness); (iv) number of years working in healthcare and (v) sentiments felt by caregivers when they are about to provide spiritual care. For a list of previous studies relevant to these factors, see [16], p. 864–865.

In the 2003 study, the concept of ‘attitudes to spiritual care’ was operationalized and addressed in terms of specific indicators of attitudes to spiritual care. These indicators were chosen because they had all functioned, either explicitly or implicitly, as indicators of attitudes to spiritual care in previous research reports identified through a literature review prior to the 2003 study (for details of this review, see [14, 16]). These indicators include (i) opinions about whether holistic care includes spirituality; (ii) opinions about whether nursing staff should be concerned with the spiritual needs of their patients; (iii) opinions about whether spiritual care are provided by the nursing staff (the colleges) on their ward; (iv) opinions about education in spiritual care; (v) the extent to which spiritual care is provided; (vi) a self‐estimation of one’s capacity to provide spiritual care and (vii) sentiments felt by caregivers when they are about to provide spiritual care. The last indicator turned out to be problematic since, in previous studies, it has been treated as a *factor influencing attitudes* to spiritual care as well as an *indicator of attitudes* to spiritual care [16]. In the 2003 study, it was analysed both as an *influencing factor* and as an *indicator of attitudes* to spiritual care. The same procedure is used in the 2023 study.

## 3. Methods

The design of the study is a quantitative, longitudinal and comparative design, as outlined in the following. Please note that the introduction and description of the study’s subjects, method and the discussions thereof follow, in part, the introduction and description of the subjects and method and discussions in [19].

### 3.1. Study Design

In order to get as comparable data as possible, an as exact as possible replication study was conducted in the 2023 study. However, because the oncological clinic that is the object of the study has undergone several organisational changes during the past 20 years and because of demographic changes in Swedish society during the same period, it was deemed necessary to implement some small changes to the design of the 2023 study. In 2003, the clinic had three inpatient wards and one very small outpatient ward. By 2023, the three inpatient wards had been merged into one large inpatient ward and the outpatient ward had grown considerably larger. The nursing staff was somewhat smaller in 2023 (compared with 2003) but included a larger proportion of registered nurses than nursing auxiliaries. The most significant change in Swedish society that influenced the design of the 2023 study is that the number of Muslims in Sweden was higher in 2023 than in 2003 [28]. This demographic change informed how organized and nonorganized religiosity was assessed in the questionnaire, where the words *mosque* and *the Koran* were included with the words *church* and *the Bible* as examples of religious institutions and religious texts, respectively. In the analyses of the 2003 study, answers were only divided into professions (registered nurses and nursing auxiliaries), whereas in the 2023 study, the answers have also been divided into the inpatient and the outpatient wards since the outpatient ward has seen substantial growth in staff and patient numbers since 2003.

A paper questionnaire and a short information letter outlining the purpose of the study were handed out to all the registered nurses and nursing auxiliaries on the inpatient and outpatient wards (*n* = 82 in 2023; for the 2003 study, *n* = 93) who were on duty during the last week of January 2023 and the first week of February 2023. The same information was also given orally to the staff at a workplace meeting. The information, both the written and the oral, stressed that participation in the study was voluntary and anonymous. The questionnaires were placed at a separate table at the entrance to the staff coffee rooms at the wards during the period and, when filled in, submitted by the participants in sealed letterboxes that were located on the wards.

### 3.2. The Questionnaire

The study’s RQs were operationalized into 17 questions with given alternatives. The questionnaire also included two open‐ended questions (presented, analysed and discussed in [19, 27]) focussing on how the respondent wished to describe the term *spiritual care* and identify any difficulties they experience in providing spiritual care. The operationalization of the RQs is outlined in the tables. The questions included in the questionnaire were informed by previous studies with similar aims and is in detail accounted for in [16]. The questions Do you consider holistic care to be important in nursing? Do you consider holistic care to be provided at your ward? Do you consider holistic care to include spirituality? Do you consider that the nursing staff should be concerned with the patients’ spiritual needs? Do you consider that the nursing staff at your ward provide spiritual care? replicate some parts of Strang, Strang and Ternestedt’s [21] questions. The questions How often do you provide spiritual care? How high do you consider your ability to provide spiritual care is? Do you feel being at ease/not at ease when you give spiritual care? Do you consider that you in your education have received education in spiritual care? Do you consider that you through your work have received education in spiritual care? are adapted from Highfield, Johnston Taylor and Amenta [29].

The questions about the respondent’s spirituality were inspired by Koenig, McCullough and Larsons [30] (*Do you believe in God? How often do you attend religious meetings?* How often do you spend time with activities like praying, meditation or reading religious literature? and by The Fetzer Institute/National Institute on Aging Working Group [31] (*Do you believe in life after death?*). These questions were designed prior to the 2000s, before the distinction between religiousness and spirituality was as distinct as it has become today (see, e.g., [2]) and mirrors what Schnell [1] labels as ‘spirituality with religion’ in contrast to ‘spirituality without religion.’

The scientific community validated the questionnaire during the 2003 study through discussions with experienced researchers in nursing science, the social science of religion and statistics (cf. [32]). The questionnaire was tested in a small pilot study in which six caregivers were asked to answer the questionnaire and provide feedback on its disposition. In light of the pilot study, minor rephrasing was made in the questionnaire to increase its clarity.

The questionnaire contained a tentative working definition of the concept of ‘spiritual care’ referred to in the introduction. The whole questionnaire (in Swedish) can be found in [14].

### 3.3. Statistical Analysis

The answers to the questionnaire were statistically analysed using the Statistical Package for the Social Sciences SPSS 29.0.1.0 (2003: SPSS 11.00), including descriptive statistics, Spearman’s rank correlations and Fischer’s exact tests. Descriptive statistics were used to summarize how the respondents’ answers were distributed across the alternatives for each question and how the answers were distributed across the nurses’ level of education and the type of ward presented in the tables of Section 4.

Cross‐tabular analyses were conducted to test for significant dependence between factors and specific indicators of attitudes to spiritual care. Since some of the expected numbers in the 5 × 5 cross tables were low (i.e., below ‘5’), conducting chi‐square tests of independence were not feasible. Consequently, Fischer’s exact test was used, grouped to 2 × 2 tables. The five alternative answers to each question were thus grouped into two alternative answers in the same way as they were grouped in the 2003 study. See respectively table for more information regarding how the answers were grouped into pairs. For example, Fischer’s exact test comparing the number of years working in healthcare for the inpatient and the outpatient wards from Table 1 was performed on the 2 × 2 table (3, 10; 0, 21); the inpatient ward had 22 respondents of which 13.6%, that is, 3 of them, had worked less than 2 years, and 13.6% + 31.8% = 45.4% (10 of them) had worked 15 years or more. Similarly, for the outpatient ward, none of the 25 respondents had worked less than two years and 20% + 64% = 84% (21 of them) had worked 15 years or more, cf. Table 1.

**TABLE 1:** Number of years working in healthcare for the respondents.

Question	Distribution of answers						
	0–2 years	3–8 years	9–14 years	15–20 years	More than 20 years	Missing answers	*n*
How many years have you worked in healthcare?							
All respondents	6.4% (7.4%)	10.6% (19.1%)	17.0% (11.8%)	17.0% (22.1%)	48.9% (39.7%)	0 (0)	47 (68)
Only registered nurses	8.6% (5.1%)	11.4% (30.8%)	20.0% (12.8%)	17.1% (20.5%)	42.9% (30.8%)	0 (0)	35 (40)
Only nursing auxiliaries	0.0% (11.1%)	8.3% (0.0%)	8.3% (11.1%)	16.7% (25.9%)	66.7% (51.9%)	0 (0)	12 (26)
The inpatient ward	13.6%	18.2%	22.7%	13.6%	31.8%	0	22
The outpatient ward	0.0%	4.0%	12.0%	20.0%	64.0%	0	25

*Note:* Two respondents have omitted to state their profession in the 2003 sample. In the cross‐tabular analysis presented in Section 4.3 related to Fisher’s exact test, comparing the inpatient and outpatient ward, the two groups ‘0–2 years’ and ‘15–20 years’ combined with ‘More than 20 years’ were used. The figures from 2023 are shown without parenthesis; the figures from 2003 are shown in parenthesis.

Fischer’s exact test was performed on every combination of the six indicators and eight factors, resulting in 48 tests and their corresponding *p* values see section 4.3. Only *p* values that were below the nominal significance level of 0.05 are reported. To adjust for the multiple tests reported in section 4.3, we applied a Bonferroni correction to identify any significant correlations, comparing the *p* values to 0.05/48 = 0.001. Fischer’s exact test was also used for some additional variables. Spearman’s rank correlations between factors and indicators, indicating the direction and strength of dependence between factors and indicators, were computed on the grouped (2‐level) answers (section 4.3).

### 3.4. Ethical Considerations

The study was approved by an ethical committee (Etikprövningsmyndigheten, Dnr 2022‐05805‐01), and approval from the head of the clinic and operation managers for the wards was secured. Participation in the study was voluntary and anonymous.

## 4. Results

In the following, when differences between data from 2003 to 2023 are reported, *p* values are generally only given when the differences are statistically significant, *p* < 0.05. Unless, otherwise, specified, all analyses were performed using Fisher’s exact test.

### 4.1. Response Rate

In the 2003 study, the total response rate was 73% (*n* = 93). The response rate varied across the four oncology wards that participated in the study. The three inpatient wards showed a response rate of 94%, 60% and 59%, respectively, and the outpatient ward a response rate of 80%. In the 2023 study, the response rate was 57% (*n* = 82), but the response rate varied between the wards. In the inpatient ward, 45% of staff responded (22 out of 49), compared with 76% in the outpatient ward (25 out of 33).

### 4.2. Descriptive Statistics of the 2003 and 2023 Studies: Similarities and Differences

Here, we describe similarities and differences found between the studies from 2003 to 2023 in closer detail. The RQ addressed in each section are stated in the headlines.

#### 4.2.1. Professional Position and Number of Years in Healthcare (RQ4c, RQ4d and RQ5)

Among the 47 respondents in the 2023 study, 74% were registered nurses and 26% were nursing auxiliaries. In the 2003 study, 57% of the 68 respondents were registered nurses and 40% were nursing auxiliaries (two respondents had omitted to state their profession). The respondents’ years of experience in healthcare were distributed similarly in both 2003 and 2023. In 2023, there was a significantly higher fraction of the outpatient staff that had worked more than 15 years compared with the inpatient ward (*p* = 0.048) (cf. Table 1).

#### 4.2.2. Belief in God and Life After Death (RQ4b and RQ5)

The proportion of staff members who reported that they believe in God decreased as a whole between 2003 and 2023 but remained the same for the nursing auxiliaries (Table 2). The proportion of staff members who reported they do not believe in God increased across every category, and the proportion of agnostics (those who report they do not know) decreased. Significant differences in belief in God (‘yes’ and ‘no’ responses) between 2003 and 2023 were found for all staff (*p* = 0.036) and registered nurses (*p* = 0.035) but not for the nursing auxiliaries. No significant differences were observed regarding belief in life after death (Table 2).

**TABLE 2:** Religious faith of the respondents.

Questions	Distribution of answers				
	Yes	No	Do not know	Missing answers	*n*
Do you believe in God?					
All respondents	40.4% (52.9%)	46.8% (23.5%)	12.8% (22.1%)	0 (1)	47 (67)
Only registered nurses	37.1% (57.5%)	48.6% (20.0%)	14.3% (20.0%)	0 (1)	35 (39)
Only nursing auxiliaries	50.0% (50.0%)	41.7% (30.8%)	8.3% (19.2%)	0 (0)	12 (26)
The inpatient ward	31.8%	63.6%	4.5%	0	22
The outpatient ward	48.0%	32.0%	20.0%	0	25
Do you believe in life after death?					
All respondents	34.0% (55.9%)	29.8% (22.1%)	36.2% (22.1%)	0 (0)	47 (68)
Only registered nurses	31.4% (65.0%)	28.6% (17.0%)	40.0% (17.0%)	0 (0)	35 (40)
Only nursing auxiliaries	50.0% (46.2%)	34.0% (30.8%)	17.0% (23.1%)	(0) 0	12 (26)
The inpatient ward	18.2%	36.4%	45.5%	0	22
The outpatient ward	48.0%	24.0%	28.0%	0	25

*Note:* Two respondents have omitted to state their profession in the 2003 sample. In the cross‐tabular analysis presented in Section 4.3 related to Fisher’s exact test, the alternatives ‘yes’ and ‘no’ were used. The figures from 2023 are shown without parenthesis; the figures from 2003 are shown in parenthesis.

In the 2023 study, responses were also analysed separately for the inpatient and outpatient wards. Among the inpatient staff, 32% reported believing in God, compared with 48% of the outpatient staff. Conversely, 64% of the inpatient staff stated that they did not believe in God, while the corresponding figure for the outpatient ward was 32%. However, these differences were not statistically significant. The distribution of responses concerning belief in life after death followed a similar pattern to the belief in God, including the changes observed between 2003 and 2023.

#### 4.2.3. Organized and Nonorganized Religiousness (RQ4b and RQ5)

The staff reported lower levels of engagement in organized religious activities in 2023 compared with 2003 (Table 3). In 2023, staff on the inpatient ward were less engaged in organized religious activities than those on the outpatient ward although not statistically significant. Differences between 2003 and 2023, as well as between the two wards, were smaller in relation to engagement in nonorganized religiousness (Table 4).

**TABLE 3:** Organized religiousness of the respondents.

Question	Distribution of answers						
	Never	At most once a year	A few times per year	Around once or twice a month	About every week	Missing answers	*n*
How often do you attend religious meetings (for example, in a church or mosque)							
All respondents	30% (22%)	45% (37%)	11% (27%)	6% (6%)	8% (7%)	0 (1)	47 (67)
Only registered nurses	29% (17%)	40% (45%)	14% (23%)	9% (5%)	9% (7%)	0 (1)	35 (39)
Only nursing auxiliaries	33% (27%)	58% (27%)	0% (31%)	0% (8%)	8% (8%)	0 (0)	12 (26)
The inpatient ward	59%	18%	14%	10%	0%	0	22
The outpatient ward	48%	20%	4%	16%	12%	0	25

*Note:* Two respondents have omitted to state their profession in the 2003 sample. In the 2003 study, the question was instead formulated like this: “How often do you go to church or attend religious meetings?” In the cross‐tabular analysis presented in Section 4.3 related to Fisher’s exact test, the alternatives ‘a few times per year,’ ‘at most once a year’ and ‘never’ have been combined as well as the alternatives ‘around once or twice a month’ and ‘about every week.’ The figures from 2023 are shown without parenthesis; the figures from 2003 are shown in parenthesis.

**TABLE 4:** Nonorganized religiousness of the respondents.

Question	Distribution of answers						
	Never	A few times per year	A few times a month	Some times a week	Every day	Missing answers	*n*
How often do you spend time with activities like praying, meditation or reading religious literature (for example, the Bible or the Koran)?							
All respondents	53% (47%)	19% (18%)	9% (6%)	13% (19%)	6% (10%)	0 (0)	47 (68)
Only registered nurses	54% (45%)	20% (17%)	6% (5%)	11% (23%)	9% (10%)	0 (0)	35 (40)
Only nursing auxiliaries	50% (50%)	17% (19%)	17% (4%)	17% (15%)	0% (12%)	0 (0)	12 (26)
The inpatient ward	45%	17%	5%	23%	10%	0	22
The outpatient ward	50%	19%	4%	15%	12%	0	26

*Note:* Two respondents have omitted to state their profession in the 2003 sample. In the 2003 study, the question was instead formulated like this: ‘How often do you spend time with activities like praying, meditation or reading the Bible?’ In the cross‐tabular analysis presented in Section 4.3 related to Fisher’s exact test, the alternatives ‘a few times per year’ and ‘never’ have been combined as well as the alternatives ‘a few times a month,’ ‘sometimes a week’ and ‘every day.’ The figures from 2023 are shown without parenthesis; the figures from 2003 are shown in parenthesis.

#### 4.2.4. Holistic and Spiritual Care (RQ1 and RQ5)

There is a high degree of similarity between 2003 and 2023 regarding the importance of holistic care in nursing (Table 5). Almost all the staff agreed that holistic care should include spirituality, a smaller proportion of the staff members thought that holistic care was provided on their ward in 2023 compared with 2003. In 2023, there is barely any difference between the inpatient and the outpatient ward in their views of the importance of holistic care, including the question whether holistic care should include spirituality.

**TABLE 5:** The respondents’ opinions about holistic care and spiritual care.

Questions	Distribution of answers						
	1	2	4	5	(Do not know)	Missing answers	*n*
Do you consider holistic care to be important in nursing?							
All respondents	0% (0%)	0% (0%)	43% (38%)	50% (47%)	7% (15%)	1 (0)	46 (68)
Only registered nurses	0% (0%)	0% (0%)	35% (37%)	56% (55%)	9% (8%)	1 (0)	34 (40)
Only nursing auxiliaries	0% (0%)	0% (0%)	67% (42%)	33% (35%)	0% (23%)	0 (0)	12 (26)
The inpatient ward	0%	0%	29%	62%	5%	0	22
The outpatient ward	0%	0%	29%	66%	8%	2	24
Do you consider holistic care to be provided at your ward?							
All respondents	2% (3%)	23% (25%)	49% (23%)	0% (9%)	26% (40%)	0 (0)	47 (68)
Only registered nurses	3% (0%)	31% (0%)	43% (37%)	0% (55%)	23% (8%)	0 (0)	35 (40)
Only nursing auxiliaries	0% (0%)	0% (0%)	67% (42%)	0% (35%)	33% (23%)	0 (0)	12 (26)
The inpatient ward	0%	32%	54%	0%	14%	0	22
The outpatient ward	4%	16%	44%	0%	36%	0	26
Do you consider holistic care to include spirituality?							
All respondents	0% (0%)	2% (3%)	38% (37%)	53% (40%)	6% (21%)	0 (0)	47 (68)
Only registered nurses	0% (0%)	3% (5%)	34% (35%)	57% (50%)	5% (10%)	0 (1)	35 (39)
Only nursing auxiliaries	0% (0%)	0% (0%)	50% (42%)	42% (27%)	8% (31%)	0 (0)	12 (26)
The inpatient ward	0%	4%	46%	46%	4%	0	40
The outpatient ward	0%	0%	32%	60%	8%	0	26

*Note:* Two respondents have omitted to state their profession in the 2003 sample. The scale is from 1 to 5, where value 1 means ‘no, not at all’ and value 5 means ‘yes, very much.’ ‘Do not know’ had the value 3 and was in the middle of the scale in the questionnaire. The figures from 2023 are shown without parenthesis; the figures from 2003 are shown in parenthesis.

#### 4.2.5. Engagement in Spiritual Care (RQ2 and RQ5)

The number of staff who are positively disposed to engagement in spiritual care increased from 65% in 2003 to 75% in 2023 (Table 6). The staff on the outpatient ward reported to a higher degree that nursing staff should be concerned with their patients’ spiritual needs compared with the staff at the inpatient ward.

**TABLE 6:** The opinion of the respondents on whether the nursing staff should engage in spiritual care.

Question	Distribution of answers						
	1	2	4	5	(Do not know)	Missing answers	*n*
Do you consider that the nursing staff should be concerned with the patients’ spiritual needs							
All respondents	0% (2%)	8% (10%)	45% (40%)	30% (25%)	17% (23%)	0 (0)	47 (68)
Only registered nurses	0% (0%)	6% (12%)	43% (35%)	37% (33%)	14% (20%)	0 (0)	35 (40)
Only nursing auxiliaries	0% (0%)	17% (8%)	50% (50%)	8% (15%)	25% (27%)	0 (0)	12 (26)
The inpatient ward	0%	18%	41%	23%	18%	0	22
The outpatient ward	0%	0%	48%	36%	16%	0	25

*Note:* Two respondents have omitted to state their profession in the 2003 sample. The scale is from 1 to 5, where value 1 means ‘no, not at all’ and value 5 means ‘yes, very much.’ ‘Do not know’ had the value 3 and was in the middle of the scale in the questionnaire. In the cross‐tabular analysis presented in Section 4.3, related to Fisher’s exact test, values 1 and 2 have been combined as well as 4 and 5. The figures from 2023 are shown without parenthesis; the figures from 2003 are shown in parenthesis.

The respondents’ estimation of whether the nursing staff should provide spiritual care (as shown in Table 7) are rather similar between 2003 and 2023.

**TABLE 7:** The respondents’ estimation of the nursing staffs providing of spiritual care.

Question	Distribution of answers						
	1	2	4	5	(Do not know)	Missing answers	*n*
Do you consider that the nursing staff at your ward provide spiritual care?							
All respondents	6% (9%)	24% (30%)	35% (22%)	0% (6%)	35% (34%)	0 (0)	47 (68)
Only registered nurses	9% (7%)	29% (33%)	35% (18%)	0% (7%)	27% (35%)	0 (0)	35 (40)
Only nursing auxiliaries	0% (11%)	8% (27%)	33% (31%)	0% (4%)	58% (27%)	0 (0)	12 (26)
The inpatient ward	5%	36%	32%	0%	27%	0	22
The outpatient ward	8%	12%	37%	0%	42%	0	25

*Note:* Two respondents have omitted to state their profession in the 2003 sample. The scale is from 1 to 5, where value 1 means ‘no, not at all’ and value 5 means ‘yes, very much.’ ‘Do not know’ had the value 3 and was in the middle of the scale in the questionnaire. In the cross‐tabular analysis presented in Section 4.3, related to Fisher’s exact test, values 1 and 2 have been combined as well as 4 and 5. The figures from 2023 are shown without parenthesis; the figures from 2003 are shown in parenthesis.

Small changes are noticeable regarding how often the respondents provided spiritual care themselves, as observed in Table 8, where the proportion of staff members who provided spiritual care often, or even daily, increased from 3% (in 2003) to 10% (in 2023). The proportion of staff members who never, or hardly never, provide spiritual care decreased in 2023. This change is more noticeable in the nursing auxiliary group. In 2003, there was a clear tendency for registered nurses to provide more spiritual care than nursing auxiliaries. This tendency was no longer evident in 2023 (Table 8). However, it was observed in 2023 that staff at the inpatient ward tended to provide spiritual care more often than those at the outpatient ward (Table 8).

**TABLE 8:** The respondents’ estimation of how often they personally provide spiritual care.

Question	Distribution of answers						
	Never	Hardly ever	Sometimes	Often	Daily	Missing answers	*n*
How often do you provide spiritual care?							
All respondents	8% (16%)	32% (38%)	49% (43%)	8% (3%)	2% (0%)	0 (0)	47 (68)
Only registered nurses	6% (5%)	31% (32%)	51% (58%)	9% (2%)	3% (0%)	0 (0)	35 (40)
Only nursing auxiliaries	17% (27%)	33% (46%)	42% (23%)	8% (4%)	0% (0%)	0 (0)	12 (26)
The inpatient ward	5%	27%	54%	9%	5%	0	22
The outpatient ward	12%	36%	44%	8%	0%	0	25

*Note:* Two respondents have omitted to state their profession in the 2003 sample. In the cross tabular analysis in Section 4.3, related to Fisher’s exact test, the alternatives ‘hardly ever’ and ‘never’ have been combined as well as the alternatives ‘sometimes,’ ‘often’ and ‘daily.’ The figures from 2023 are shown without parenthesis; the figures from 2003 are shown in parenthesis.

As seen in Table 9, the respondents’ self‐estimated ability to provide spiritual care increased slightly in 2023 compared with 2003. The staff on the outpatient ward reported that they provided spiritual care more often than the staff on the inpatient ward.

**TABLE 9:** The respondents’ self‐estimated ability to provide spiritual care.

Question	Distribution of answers						
	Very low	Low	Acceptable	High	Very high	Missing answers	*n*
How high do you consider your ability to provide spiritual care is?							
All respondents	8% (18%)	32% (32%)	49% (44%)	8% (4%)	2% (0%)	0 (1)	47 (67)
Only registered nurses	6% (7%)	31% (35%)	51% (50%)	9% (5%)	3% (0%)	0 (1)	35 (39)
Only nursing auxiliaries	17% (27%)	33% (31%)	42% (39%)	8% (4%)	0% (0%)	0 (0)	12 (26)
The inpatient ward	9%	32%	46%	9%	4%	0	22
The outpatient ward	8%	24%	48%	16%	4%	0	25

*Note:* Two respondents have omitted to state their profession in the 2003 sample. In the cross‐tabular analysis in Section 4.3, related to Fisher’s exact test, the alternatives ‘low’ and ‘very low’ have been combined as well as the alternatives ‘acceptable,’ ‘high’ and ‘very high.’ The figures from 2023 are shown without parenthesis; the figures from 2003 are shown in parenthesis.

#### 4.2.6. Education in Spiritual Care (RQ3, RQ4a and RQ5)

Overall, education in spiritual care was considered to be more important in 2023 than in 2003 (Table 10). This difference was more pronounced for registered nurses than for nursing auxiliaries. Moreover, staff on the outpatient ward considered education in spiritual care to be more important than staff on the inpatient ward, see Table 10.

**TABLE 10:** The respondents’ opinions about education in spiritual care.

Questions	Distribution of answers						
	1	2	4	5	(Do not know)	Missing answers	*n*
Do you consider education in spiritual care to be important?							
All respondents	0% (4%)	6% (3%)	38% (46%)	49% (25%)	6% (22%)	0 (0)	47 (68)
Only registered nurses	0% (2%)	9% (3%)	37% (48%)	54% (27%)	0% (20%)	0 (0)	35 (40)
Only nursing auxiliaries	0% (0%)	0% (4%)	42% (46%)	33% (23%)	25% (27%)	0 (0)	12 (26)
The inpatient ward	0%	14%	32%	45%	9%	0	22
The outpatient ward	0%	0%	44%	52%	4%	0	25
Do you consider that you in your education have received education in spiritual care?							
All respondents	25% (38%)	49% (34%)	15% (22%)	2% (2%)	9% (4%)	0 (0)	47 (68)
Only registered nurses	26% (20%)	49% (45%)	17% (30%)	3% (0%)	6% (5%)	0 (0)	35 (40)
Only nursing auxiliaries	25% (62%)	50% (19%)	8% (11%)	0% (4%)	17% (4%)	0 (0)	12 (26)
The inpatient ward	36%	46%	9%	0%	9%	0	22
The outpatient ward	16%	52%	20%	4%	8%	0	25
Do you consider that you through your work have received education in spiritual care?							
All respondents	30% (31%)	40% (35%)	15% (22%)	4% (0%)	11% (12%)	0 (0)	47 (68)
Only registered nurses	34% (2%)	46% (3%)	9% (48%)	6% (27%)	6% (20%)	0 (0)	35 (40)
Only nursing auxiliaries	17% (0%)	25% (4%)	33% (46%)	0% (23%)	25% (27%)	0 (0)	12 (26)
The inpatient ward	41%	18%	23%	4%	14%	0	22
The outpatient ward	20%	60%	8%	4%	18%	0	25

*Note:* Two respondents have omitted to state their profession in the 2003. The scale is from 1 to 5, where value 1 means ‘no, not at all’ and value 5 means ‘yes, very much.’ ‘Do not know’ had the value 3 and was in the middle of the scale in the questionnaire. In the cross‐tabular analysis in Section 4.3, related to Fisher’s exact test, the values 1 and 2 have been combined as well as 4 and 5 for the first and second questions, and the third question was not analysed in cross‐tabular tests. The figures from 2023 are shown without parenthesis; the figures from 2003 are shown in parenthesis.

Fewer staff members claimed to have received education in spiritual care through their formal education or work in 2023 compared with 2003 (Table 10). At the outpatient ward (2023), staff tended to have received more education in spiritual care during their education than at the inpatient ward. The opposite pattern was observed (for the outpatient and inpatient wards) regarding education in spiritual care at work (Table 10).

#### 4.2.7. Sentiments of Being at Ease While Providing Spiritual Care (RQ4e and RQ5)

There are some changes between 2003 and 2023 regarding the sentiments of being at ease if/when providing spiritual care overall. The nursing auxiliaries reported in 2023 that they were more at ease or well at ease when providing spiritual care, both compared with the nursing auxiliaries in 2003 and with the registered nurses 2023 (Table 11). At the outpatient ward (2023), they felt more at ease providing spiritual care compared with the inpatient ward (but not significantly so).

**TABLE 11:** The respondents’ sentiment of being at ease/not at ease if/when providing spiritual care.

Question	Distribution of answers						
	Very much not at ease	Not at ease	Neither nor	At ease	Well at ease	Missing answers	*n*
Do you feel being at ease/not at ease if/when you give spiritual care?							
All respondents	0% (4%)	4% (7%)	51% (43%)	36% (41%)	9% (2%)	0 (0)	47 (68)
Only registered nurses	0% (3%)	6% (8%)	51% (40%)	31% (48%)	11% (0%)	0 (0)	35 (40)
Only nursing auxiliaries	0% (4%)	0% (8%)	50% (46%)	50% (35%)	0% (4%)	0 (0)	12 (26)
The inpatient ward	0%	5%	64%	27%	5%	0	22
The outpatient ward	0%	4%	40%	44%	12%	0	25

*Note:* Two respondents have omitted to state their profession in the 2003 sample. In the cross‐tabular analysis in Section 4.3, related to Fisher’s exact test, the alternatives ‘very much not at ease’ and ‘not at ease’ have been combined as well as the alternatives ‘at ease’ and ‘well at ease.’ The figures from 2023 are shown without parenthesis; the figures from 2003 are shown in parenthesis.

### 4.3. Factors and Indicators of Attitudes to Spiritual Care in 2003 and 2023: Similarities and Differences (RQ5)

Table 12 shows the Spearman correlations between factors and indicators for attitudes to spiritual care. Positive values indicate a positive dependence, and negative values indicate a negative dependence. The *p* values from Fisher’s exact test were used to test for significant dependency between different factors and indicators. Because of the large number of tests that were performed, that is, forty eight, the significance must be interpreted with caution.

**TABLE 12:** Spearman correlations between factors influencing attitudes to spiritual care and indicators for attitudes to spiritual care, previously identified through a literature review preceding the 2003 study *p* values from Fisher’s exact test, testing if a factor correlated with an indicator to spiritual care, are presented below each correlation.

Indicators	Factors							
	Nonorganized religiousness	Belief in god	Belief in life after death	Organised religiousness	Degree of education (profession)	Opinions about if one in ones’ education has received education in spiritual care	Sentiments of being at ease if/when providing spiritual care	Work years in healthcare
The opinion if nursing staff should be concerned with patients’ spiritual needs		0.344^∗^ 0.046						
							(0.610^∗∗^ 0.014)	

The extent to which nursing staff at the respondents’ own ward is considered to perform spiritual care								
	(−0.117 0.037)	(−0.388^∗^ 0.003)	(−0.379^∗^ 0.024)					

The extent to which education in spiritual care is considered to be important								
			(0.048 0.173)				(0.454^∗∗^ 0.006)	

How often the respondent provides spiritual care	0.316^∗^ 0.046							
	(0.285^∗^ 0.002)	(0.212 0.031)			(−0.354^∗∗^ 0.019)		(0.596^∗∗^ 0.000^a^)	

The self‐estimated ability to provide spiritual care	0.465^∗∗^ 0.001^a^	0.440^∗∗^ 0.008	0.437^∗^ 0.023	0.315^∗^ 0.039				
	(0.332^∗∗^ 0.027)			(0.393^∗∗^ 0.001^a^)		(0.418^∗∗^ 0.023)	(0.695^∗∗^ 0.000^a^)	

Sentiments of being at ease if/when providing spiritual care								
	(0.335^∗∗^ 0.034)	(0.381^∗∗^ 0.004)						

*Note:* The figures from 2023 are given without parenthesis (upper two rows for each indicator), and the figures from 2003 are given in parenthesis (lower two rows för each indicator). Only the figures with *p* values from Fisher’s exact test below 0.05 are presented.

^a^Significant dependence between factor and indicator at overall 0.05 significance level after adjusting for the 48 tests using Bonferroni correction. Note that the factors ‘belief in God’ and ‘belief in life after death’ have been given the value 1 for ‘no’ and 2 for ‘yes’ and the factor ‘degree of education’ has been given the value 1 for nursing auxiliaries and 2 for registered nurses when performing Spearman’s test.

^∗^Correlation is significant at the nominal 0.05 level (2‐tailed).

^∗∗^Correlation is significant at the nominal 0.01 level (2‐tailed).

‘Nonorganized religiousness’ and ‘the sentiment of being at ease/not at ease if/when providing spiritual care’ were the factors that correlated significantly with the largest number of indicators in the 2003 questionnaire. The factors ‘belief in God’ and ‘belief in life after death’ also significantly correlated with several indicators. The respondents’ reported engagement in ‘organized religiousness,’ their ‘profession’ and their ‘perceived degree of education in spiritual care’ significantly correlated with one indicator each. In 2023, the factors ‘nonorganized religiousness’ and ‘belief in God’ significantly correlated with two indicators each, while the factors ‘belief in life after death,’ ‘organized religiousness’ and ‘profession’ were significantly correlated with one indicator each. The correlations between factors and indicators were all statistically significant at a nominal level of *p* < 0.05. However, after adjusting for the multiple (forty eight) tests that were performed (as per Table 12), using the Bonferroni correction, significant dependence at the overall 0.05 level in 2003 was found between:

i. The factor ‘sentiments of being at ease if/when providing spiritual care’ and the indicator ‘how often the respondent provided spiritual care’
ii. The factor ‘sentiments of being at ease if/when providing spiritual care’ and the indicator ‘the self‐estimated ability to provide spiritual care’
iii. The factor ‘organized religiousness’ and the indicator ‘the self‐estimated ability to provide spiritual care.’

In 2023, a significant dependence was found between the factor ‘nonorganized religiousness’ and the indicator ‘the self‐estimated ability to provide spiritual care.’

## 5. Discussion

### 5.1. On the Results

#### 5.1.1. Response Rate

The low response rate in 2023 from the inpatient ward could be attributed to the fact that the participants filled in the questionnaire when they were at work. The workload on the inpatient ward at the time of study was higher than on the outpatient ward (according to the operation managers). However, it is also possible that a potential bias may have existed since we do not know if those staff members who filled in the questionnaire were those staff members who held the strongest opinions about spiritual care (whether their opinion was for or against spiritual care).

#### 5.1.2. Differences in Inpatient and Outpatient Wards

The differences between the inpatient and outpatient wards in 2023, with respect to religious belief and organized and nonorganized religiousness, can probably be explained by the considerable difference in the number of years worked in healthcare between the staff on the two wards. On the outpatient ward, 64% had worked 20 years or more in healthcare, whereas the corresponding number on the inpatient ward was 32%. In this respect, the results of the outpatient ward are more similar to the results of the 2003 study than the inpatient ward’s results in 2023.

#### 5.1.3. Attitudes to Spiritual Care From a Holistic Care Perspective

For the 2003 study, Lundmark [16] concluded that the results of the study and those of Strang et al.’s study [21] confirmed each other to a great extent, especially with regard to attitudes to spiritual care from a holistic care perspective. According to the nursing staff’s attitudes to holistic care, (i) holistic care was desirable in the Swedish healthcare context, (ii) holistic care should include the spiritual needs of the patients and (iii) holistic care had not yet been realized in the Swedish healthcare system ([16], p. 872). The results of the 2023 study revealed a similar picture, where the results indicate that holistic healthcare seems to have become more integrated in nursing work at the clinic during the 20 years that had passed since the 2003 study but has yet not been fully realized (Table 5).

#### 5.1.4. Providing Spiritual Care

Despite the increase of respondents reporting that the nursing staff should be concerned with their patients’ spiritual needs from 2003 to 2023 (Table 6), not much differences can be seen between 2003 and 2023 with regard to the respondents’ estimation of the frequency by which nursing staff provide spiritual care (Table 7) or with regard to the self‐estimated ability of individual staff members to provide spiritual care (Table 9). This observation is somewhat surprising since the respondents who reported that they sometimes, often or daily provided spiritual care increased from 46% in 2003 to 59% in 2023 (Table 8). A possible explanation for this apparent discrepancy may be found in the division of the respondents in terms of their roles as nursing auxiliaries and registered nurses. The 2003 study demonstrated a significant disparity between nursing auxiliaries and registered nurses, with a markedly higher proportion of registered nurses (60%) reporting the provision of spiritual care sometimes, often or daily compared with nursing auxiliaries (25%). This was surprising since no distinctive differences in background factors, including their ‘belief in God’ and ‘nonorganized religiousness’ existed between the nursing auxiliaries and registered nurses. Lundmark [16] argued that this discrepancy was a result of the nursing auxiliaries not being used to reflecting upon their nursing practice since the registered nurses were responsible for the documentation of the nursing interventions that took place on the ward. Consequently, this may have made the registered nurses more skilled than the nursing auxiliaries in analysing and formulating their reports on their different nursing interventions. This difference was substantially smaller in the 2023 study. It may be the case that the nursing auxiliaries at the oncology clinic are nowadays much more active in producing documentation than they were 20 years ago, suggesting that they have acquired the skill to reflect upon their nursing interventions.

#### 5.1.5. Education in Spiritual Care

In 2003, education in spiritual care was deemed important, but even more so in 2023 (Table 10). This is reasonable, given that holistic care was more important in 2023 than in 2003. Furthermore, in 2023, holistic care was still considered to include spiritual care (Table 5). This observation is contrasted by a considerably smaller number of the 2023 staff, especially among the registered nurses, who reported that they had received education in spiritual care during their formal education. A considerably larger proportion of the outpatient staff compared with the inpatient staff reported that they had received education in spiritual care during their formal education. This suggests that the staff members who had worked longer in healthcare and likely have an older education claimed to have received more education in spiritual care than those who had a more recent education. To what extent this relates to the accounted for changes on how spiritual care is addressed in textbooks on palliative care is still an open question. But the recent Swedish trend to withdraw the concept of spirituality from what palliative care should address may not to be well anchored in the nursing staffs’ perceptions.

#### 5.1.6. Feeling at Ease Giving Spiritual Care

In 2023, we note a considerable difference between the two wards, where far more of the staff on the outpatient ward felt at ease or even well at ease when giving spiritual care. In this respect, the outpatient ward in 2023 mirrors the whole clinic in the 2003 study. Why this was the case may be attributed to the difference between the inpatient and outpatient wards in terms of staff experience? Recall that, in 2023, two‐thirds (64%) of the outpatient staff had worked for the healthcare system before 2003. The corresponding proportion for the staff on the inpatient ward was almost one‐third (32%). We suggest there are at least three different possible reasons for this difference between the two wards: (i) it might reflect feelings that were already present in the staff when they were working in 2003, which, if that was the case, have not changed for this group during the intervening 20 years, or (ii) it could be that with age and experience, a nurse’s feelings of being at ease when providing spiritual care increases. Finally, (iii) it could also be because of the higher proportion of outpatient staff who reported that they engaged in religious activities (Table 3).

#### 5.1.7. Discrepancies in Understandings of What Spiritual Care Is

A somewhat unexpected finding in the 2003 study is the negative Spearman correlations associated with the indicator ‘the extent to which nursing staff on the respondents’ ward are considered to provide spiritual care (Table 12). The correlations identified in the 2003 study suggest that those staff members who reported ‘belief in God’ and ‘belief in life after death’ or scored high on ‘nonorganized religiousness’ held a narrower understanding of what spiritual care is when compared with the rest of the staff. These negative correlations were not found in the 2023 study, thereby supporting the interpretation of the 2003 study since the number of staff members who reported ‘belief in God’ or ‘belief in life after death’ decreased between 2003 and 2023.

#### 5.1.8. Factors Correlating With Attitudes to Spiritual Care

Making further comparisons between the 2003 and 2023 studies, the factors ‘nonorganized religiousness’ and ‘belief in God’ can be viewed as being more stable over time than the other factors since only they were correlating with indicators for attitudes to spiritual care in both 2003 and 2023 (Table 12). Concerning the other factors, the differences between the 2003 study and the 2023 study might be seen as supporting the claims that the factors that correlate with attitudes to spiritual care can change over time and that some of the factors in the 2003 study were of less relevance in 2023 (Table 12).

When we compare the dependencies between factors and indicators for attitudes to providing spiritual care in the 2003 study and the 2023 study, one conclusion is that ‘the self‐estimated ability to provide spiritual care’ is the only indicator that significantly correlates with factors in both the 2003 study and the 2023 study. Consequently, from a longitudinal perspective, this indicator seems to be the most stable indicator for attitudes to spiritual care over time and, therefore, the most promising to use in longitudinal studies on attitudes to spiritual care.

#### 5.1.9. Determinants to Attitudes of Spiritual Care Over Time

The factor ‘organized religiousness’ (but not ‘nonorganized religiousness’) was significantly correlated with ‘the self‐estimated ability to provide spiritual care’ in the 2003 study, whereas in the 2023 study, ‘nonorganized religiousness’ (not ‘organized religiousness’) was significantly correlated with the same indicator. This difference might mirror the decrease of organized religiousness in today’s Swedish population and, thus, illustrates that attitudes to spiritual care can indeed be subject to changing influences though there can of course be alternative explanations for these changes. However, the results of this study provide us with reasons to believe that at least some determinants of the nurses’ spiritual care practices (including the influencing factors investigated in this study) are not stable over time and a possible reason for this can be attributed to societal changes.

#### 5.1.10. Identification of Factors Influencing Spiritual Care

As stated in the introduction, the identification of factors that may influence spiritual care is important, especially if we wish to learn how to allocate resources to better meet the spiritual needs of patients. In the 2023 study, the only statistically significant factor correlated with attitudes to spiritual care was ‘nonorganized religiousness.’ This result is consistent with other studies reporting nurses’ own spirituality as a facilitator for spiritual care [12]. As a potential resource that can be used to meet the spiritual needs of patients, this factor probably lies outside the scope of interventions or educational directives that might be initiated by a healthcare institution (whether it be through education or through the daily work that is done on the ward), at least in a secular country like Sweden, where most healthcare is provided in a nonconfessional setting and religiosity is seen as something particularly private [26].

### 5.2. On Limitations, Strengths and Future Research

The design of this study only permits conclusions to be drawn about dependencies between factors and indicators, not about causality. Problems concerning the identification of causes and effects in the material were discussed in Lundmark [16] using the example ‘sentiments of being at ease/not at ease if/when providing spiritual care.’ The literature study that preceded the 2003 study demonstrated that these sentiments can be treated both as a factor influencing attitudes to spiritual care and as an indicator of attitudes to spiritual care. In the 2003 study and the 2023 study, these sentiments were considered from both perspectives. In the 2003 study (though not in the 2023 study), it was clear that these sentiments were both influenced by factors of attitudes to spiritual care and influenced other indicators of attitudes to spiritual care, an observation also done in other studies on determinants to spiritual care [12]. The relationships between the factors and indicators presented in this study are complex. Consequently, a more extensive study should be conducted to clarify these connections and relationships.

The indicators used in this study were initially identified as part of a review of the results of international research that was conducted over 20 years ago. Given this background and as suggested by the results of the present study, some of the indicators might be outdated. Perhaps alternative indicators of attitudes to spiritual care might better fit today’s social conditions. Furthermore, it is possible that factors specific to Swedish conditions and specific to the 2020s may influence attitudes to spiritual care.

The 2023 study followed the same operationalizing of the concepts as used in the 2003 study. This approach was chosen to maximize the comparability between the collected data used in the two studies. However, some of these concept operationalizations can be regarded as outdated today, at least the operationalization of ‘nonorganized religiosity’ into not only prayers and reading religious literature but also into meditation (Table 4). Such a perspective is problematic today because meditation is not necessarily associated with religiosity [33].

Another limitation due to the study being a replication study is, as noted in Section 3.2, that the distinction between religiousness and spirituality was not as distinct in 2003 as it is 2023. This becomes evident in how the background information on the participants own spirituality was assessed in the 2003 study where Lundmark primarily addressed what Schnell [1] labelled ‘spirituality with religion,’ omitting what she calls ‘spirituality without religion.’ If the 2023 study only focused on how nursing staff understand spiritual care in 2023, it would add valuable information to include background questions in the questionnaire that also assessed spirituality without religion. Since the focus primarily is on changes in the attitudes to spiritual care over time, the importance of being as faithful as possible to the original study design was deemed as most important, perhaps at the price of a shortage on background information concerning the informant’s eventual spirituality without religion. However, the relevant information to compare the studied oncology clinic at 2003 and 2023 with the relevant waves of WVS is precent since WVS, when trying to measure secular values, assessed what, with Schnell’s terminology, would be labelled ‘spirituality with religion.’

Given the nature of this study, small as it is in numbers of participants, its strength lies in its longitudinal case‐study design. The relevance of a case study can be measured in terms of its transferability, referring to the idea that the reader can transfer knowledge generated from one case to another similar case [34]. A criterion for validity, originally stipulated for qualitative research, but which is highly relevant also for this study, is to ensure that the study has sufficient breadth and depth to give additional insight into the issue that is being researched [35]. In addition to providing methodological information and a detailed account of the study’s results, the study’s depth was ensured by providing a sufficient background information about the clinic, educational changes and some relevant information from the WVS as it pertained to the Swedish context. In doing this, we hope that the reader has been provided with sufficient information to make their own comparisons with other cases situated in other contexts with its eventual similarities and/or differences compared with the Swedish case (see also [34]).

## 6. Conclusions

The aims of the study were to replicate the 2003 study (Aim 1) and to perform a comparative analysis of the results of the 2003 study with the replication study conducted in 2023 (Aim 2). Five RQs (RQ1–5) were posed. Concerning RQ1 and RQ5, the staff at the studied oncology clinic expresses positive attitudes to spiritual care in both studies. More nursing staff considered patients’ spiritual needs a concern in 2023. Concerning RQ2 and RQ5, there were no big differences in self‐estimated frequencies of how much the colleagues provide spiritual care, the self‐estimated ability to provide spiritual care or the self‐estimated frequency to provide spiritual care. Concerning RQ3 and RQ5, education in spiritual care was considered important in both studies but the received education in spiritual care was perceived as decreased in 2023. Concerning RQ4 and RQ5, several of the influencing factors of 2003 have lost their relevance in 2023 but ‘belief in God’ and ‘nonorganized religiousness’ are remaining stable factors over time. The factor ‘organized religiousness’ significantly correlates with the indicator ‘self‐estimated ability to provide spiritual care’ in 2003, whereas ‘nonorganized religiousness’ significantly correlates with the same indicator in 2023.

The results of the RQs indicate that some determinants of nurses’ attitudes to spiritual care remain stable over time while others do not. Changes in a determinant may be attributed to it being sensitive to societal changes, for example, a decline in religiosity or an increase in self‐expression values. This entails that, in addition to considering cultural and other contextual differences when evaluating the determinants of nurses’ attitudes to spiritual care, the determinant’s stability over time should be accounted for. This approach demands that the researcher is sensitive to the historical context of the object of study and societal or educational changes that may impact the object of study.

## Funding

No funding was received for this manuscript.

## Conflicts of Interest

The authors declare no conflicts of interest.

## Data Availability

The data that support the findings of this study are available from the corresponding author upon reasonable request.
